# Web-based tool for calculating field-specific nutrient management for rice in India

**DOI:** 10.1007/s10705-018-9959-x

**Published:** 2018-10-22

**Authors:** Sheetal Sharma, P. Panneerselvam, Rowena Castillo, Shriram Manohar, Rajendran Raj, V. Ravi, Roland J. Buresh

**Affiliations:** 1 International Rice Research Institute, New Delhi, India; 2 International Rice Research Institute, DAPO Box 7777, Metro Manila, Philippines; 3 Coconut Research Station, Tamil Nadu Agricultural University, Veppankulam, Thanjavur, Tamil Nadu, India; 4 Tamil Nadu Rice Research Institute, Tamil Nadu Agricultural University, Aduthurai, Thanjavur, Tamil Nadu, India

**Keywords:** Decision support tool, Nutrient Manager, Rice, Fertilizer recommendation, On-farm research, Nutrient balance

## Abstract

Site-specific nutrient management (SSNM) can be an alternative to a recommendation for uniform fertilizer use across a rice (*Oryza sativa* L.) production system within a country or region of a country. We developed a web-based decision support tool named Nutrient Manager for Rice (NMR), which used principles of SSNM to calculate fertilizer N, P, and K rates for individual fields based on a target yield set for each field. It also used expected growth duration of the rice variety, crop establishment method, and age of transplanted seedlings to calculate days after rice establishment for each of three applications of fertilizer N. NMR enabled P rates to match estimated removal of P with harvested grain and crop residue for the target yield set for each field. We compared field-specific fertilizer recommendations from NMR with uniform application of fertilizer provided by an existing blanket fertilizer recommendation for irrigated inbred rice (BFR) and farmer’s fertilizer practices (FFP) in on-farm trials conducted in 74 irrigated rice fields across three growing seasons in the Cauvery Delta, Tamil Nadu, India. Grain yield was 0.6–0.7 Mg ha^−1^ higher (*P* ≤ 0.05) with NMR than FFP in two of the three seasons, even though total fertilizer cost was comparable or less with NMR. Yield was comparable for NMR and BFR, but NMR reduced fertilizer N and P rates and total fertilizer cost compared to BFR. Use of NMR rather than BFR also had less risk of financial loss for a farmer. The likelihood of financial loss with a switch from FFP to BFR averaged 31%. It reduced to 18% with a switch from FFP to NMR. NMR facilitated the calculation of field-specific fertilizer N, P, and K management practices, which increased fertilizer use efficiency without loss in rice yield compared to a recommended uniform fertilizer management across fields.

**Abbreviations**
BFR: Blanket fertilizer recommendation
FFP: Farmer’s fertilizer practice
FN: Fertilizer N rate
GY: Grain yield
K: Potassium
N: Nitrogen
NMR: Nutrient Manager for Rice
P: Phosphorus
PFP: Partial factor productivity of added N
SSNM: Site-specific fertilizer management

## Introduction

The management of fertilizers can be particularly critical for profitable rice farming in Asia because fertilizers are typically the second largest input cost after labor. A study in seven major irrigated rice areas in six Asian countries showed fertilizers represented 11–28% of the annual costs of farmers for producing rice (Moya et al. 2004). Fertilizers must be applied at appropriate times and rates for efficiently increasing yield per unit of nutrient applied. Substantial portions of added fertilizer N can be lost from rice soils as gases through ammonia volatilization and nitrification–denitrification, especially when fertilizer N is not applied at rates and times synchronized with the demand of the rice crop for supplemental N (Buresh et al. 2008).

Rice fields in Asia tend to be small (often less than one hectare) and frequently variable in soil nutrient status; yet rice farmers in Asia are often provided with a recommendation using a uniform blanket dose of fertilizer N, P, and K for all fields within a geographical or administrative area. Such recommendations ignore variations across fields in supply of essential nutrients from the soil (Dobermann et al. 2003) and variations in crop management practices, which can influence the needs of the rice crop for fertilizer and the yields obtained in farmers’ fields (Witt et al. 2007). The use of blanket nutrient recommendations across a rice production area can lead to low nutrient use efficiencies and leakages of nutrients to the environment (Singh et al. 2015).

The management of fertilizers for high yields and higher efficiency of nutrient use in rice production could consequently benefit from a cost-effective, rapid, and easy-to-use approach to handle the field-specific needs of a rice crop. Site-specific nutrient management (SSNM) as developed and refined through years of research (Dobermann et al. 2002, 2004b) provides principles, which could be used to calculate field-specific management practices for fertilizer N, P, and K (Buresh et al. 2010). Fertilization using SSNM has been shown to increase yield and income in rice production (Dobermann et al. 2004b; Witt et al. 2007) and provide environmental benefits by reducing N losses and greenhouse gas emissions (Pampolino et al. 2007). The SSNM approach adjusts inputs of fertilizers based on a supply of indigenous nutrients originating from soil, plant residues, manures, and irrigation water. With SSNM, the timing and rates of fertilizer N are dynamically adjusted to match specific needs of the rice variety, field, and season (Peng et al. 2010; Witt et al. 2007).

The International Rice Research Institute in collaboration with national partners across Asia developed a web-based decision support tool named Nutrient Manager for Rice (NMR), which uses the principles of SSNM to calculate field-specific fertilizer N, P, and K recommendations. NMR is accessible through the web browser of a smartphone, tablet, or personal computer (Buresh et al. 2014). Extension workers and researchers with a mobile devise or computer can use NMR to interview a rice grower before crop establishment using 15–20 interactive questions about a specific rice field. Through this interview, information on size of the field, rice variety, sowing date, crop establishment method, and past rice yield are obtained from the rice grower. NMR uses this information to calculate a target yield of rice for the field in the upcoming cropping season and then calculate amounts and times for application of fertilizer sources for the size of the field indicated by the grower. The output of NMR can be printed and provided as a personalized one-page recommendation to the rice grower. NMR calculates fertilizer P and K rates based on field-specific nutrient input–output balances for a target yield (Buresh et al. 2010). NMR calculates fertilizer N rate based on the target yield (Buresh 2015) and distributes the N in split doses at critical growth stages (Peng and Cassman 1998; Witt et al. 2007).

The SSNM-based algorithms within NMR must be parameterized to local rice-growing conditions and then verified before a country or administrative region (such as a state in India) can confidently release NMR for wide scale use by extension services and farmers. Results from past on-farm evaluations of SSNM (Nagarajan et al. 2004; Rajendran et al. 2010) and nutrient omission plot technique trials (Dobermann et al. 2003) in the Cauvery Delta, Tamil Nadu, India enabled the development of a version of NMR parameterized to rice-growing conditions in the Cauvery Delta. Tamil Nadu is a major rice-producing state of India with much of its rice produced in the Cauvery Delta, often through intensive cropping with two irrigated rice crops per year (Nagarajan et al. 2004). Such intensive cropping with more than one rice crop per year extends across millions of hectares in Asia (GRiSP 2013).

The benefits of SSNM-based fertilizer management relative to farmers’ fertilizer practices have already been shown in early evaluations of SSNM (Dobermann et al. 2002, 2004b) and recent evaluations of decision support tools for rice (Saito et al. 2015; Banayo et al. 2018). These studies did not compare SSNM-based fertilizer management to an existing fertilizer recommendation. We hypothesized that field-specific fertilizer management, as calculated by NMR using SSNM principles, could increase efficiency of fertilizer use relative to a recommended fertilizer practice that did not consider spatial variation in rice management among fields. Our objective was to compare field-specific fertilizer management calculated by NMR with a uniform fertilizer application provided by a blanket fertilizer recommendation from Tamil Nadu Agricultural University (TNAU 2016) (BFR) and the farmer’s fertilizer practices (FFP) in the Cauvery Delta. The BFR, accessible through a website of TNAU, recommended fixed rates of N, P, and K for irrigated, high-yielding, inbred rice in the Cauvery Delta. Our study helps identify how use of precise field-specific fertilizer management for small landholdings (NMR) rather than a uniform fertilizer application across a rice production system (BFR) might affect rice farmers and rice production.

## Materials and methods

### Study area

The study was conducted in the fields of 74 rice farmers across three rice-growing seasons during 2014 and 2015 in Thanjavur District—an important irrigated rice production area in the Cauvery Delta. The study area included both the Old Delta with heavy-textured (clay loam to clay) soils and the New Delta with lighter textured (sandy loam to clay loam) soils and good drainage (Nagarajan et al. 2004). Soils in the Cauvery Delta are generally relatively low in organic C and available N, medium in available P and K (Nagarajan et al. 2004), and low in extractable zinc (Savithri et al. 1999). The climate is tropical with annual mean rainfall of 1020 mm. The majority of the rain is received through the northeast monsoon in October to early December. A map of the study site is provided by Nagarajan et al. (2004).

The major irrigated rice-growing seasons are kuruvai, samba, and thaladi. The kuruvai season from June to September is a pre-monsoon dry season with shortduration rice varieties (105-110 days) and potential to achieve high yields due to favorable temperatures and solar radiation. Thaladi and samba are rainy seasons with lower yield potential. Medium-duration rice (125–135 days) is grown during the thaladi season from October to February as a second crop after the kuruvai season. In samba, only one rice crop per year is grown, usually from August to January.

### Operation of NMR

Before the start of field trials, each selected rice farmer was interviewed by a researcher using the interactive questions of NMR, accessible through the web browser (http://webapps.irri.org/in/tn/nmr) of a smartphone or personal computer. During the interview, information was collected from each farmer on size and location of the field, season, source of irrigation water, rice variety, crop establishment method (i.e., transplanted, wet seeded, or dry seeded), and approximate rice yield in previous seasons. Each farmer was also asked to select preferred fertilizer sources for P and K from a menu of locally available NPK-, NP-, P-, and K-containing fertilizer sources. NMR did not ask about a preferred source of fertilizer N because urea was known to be used by farmers.

The collected information was transmitted from the smartphone or personal computer of the researcher via the Internet to a cloud-based server where NMR used the information to calculate a target yield and then calculate SSNM-based amounts of the farmer-selected fertilizer sources and urea required to achieve the target yield for the size of the field indicated by the farmer. The target yield, used to calculate fertilizer rates, was automatically computed by NMR using previous rice yield reported by the farmer during the interview with NMR (i.e., reported historical yield) and information from local rice experts and past field trials on yield achieved in the Cauvery Delta by the farmer-selected rice variety (i.e., baseline varietal yield). The target yield was normally set higher than the historical yield reported by a farmer to enable the farmer to achieve higher yield and net income through more efficient management of fertilizer. Detailed information on the setting of target yield is provided in Online Resource 1—Part 1.

NMR used a yield gain approach to calculate fertilizer N rate for a target yield and a nutrient input–output balance approach to calculate fertilizer P and K rates for a target yield (Buresh et al. 2010). They are explained in Online Resource 1—Part 2. NMR used information on growth duration of the selected rice variety, crop establishment method, and age of seedlings for transplanted rice to automatically calculate the number of days after crop establishment corresponding to three critical growth stages for application of fertilizer N. NMR then distributed the calculated total amount of fertilizer N into separate doses on the three calculated dates. Online Resource 1—Part 3 explains how NMR calculated the number of days after crop establishment for each application of fertilizer N.

Upon completion of calculations in the cloud-based server of NMR, the calculated amounts of fertilizer for the size of the field indicated by the farmer and times for application were transmitted via the Internet to the device of the interviewer for printing as a field-specific fertilizer recommendation (Buresh et al. 2014). The recommendation included a table reporting number of dates after crop establishment for application of exact amounts of urea and P- and K-containing fertilizer sources selected by the farmer. The recommendation was personalized to indicate the name and location of the farmer, target yield, growing season, and year. Examples of recommendations are given in Online Resource 1—Part 4.

### Treatments and experimental details

The NMR recommendation was compared with FFP and BFR in 74 trials (14 during kuruvai, 40 during samba, and 20 during thaladi) in fields of the selected rice farmers. Each field had one on-farm trial with three unreplicated treatments (NMR, FFP, and BFR) randomly assigned to plots at least 300 m^2^ in size and surrounded by an earthen levee. Each on-farm trial served as a replicate. All trials in kuruvai had irrigation from a tube well. All trials in samba relied at least partly on gravity irrigation from canals, and all trials in thaladi relied on water from rain and canal irrigation. Soil properties for field trials are reported in Online Resource 2—Part 1.

For NMR the sources, rates, and times for fertilizer (N, P, and K) application were based on the NMR recommendation, which was unique for each of the 74 field trials. The typical distribution for fertilizer N was 24% as early within the first 11 days after crop establishment, 38% at active tillering, and 38% at panicle initiation. The distribution for fertilizer N was changed to three equal doses when the estimated date of panicle initiation was < 30 days after crop establishment. All P and 50–58% of total K was applied along with early N within 11 days of crop establishment. The remaining K was applied at panicle initiation.

NMR converted the rates of N, P, and K for each application into amounts of fertilizer sources. The N at tillering and panicle initiation was always applied as urea, and K at panicle initiation was always applied as muriate of potash (KCl). The P and K in the early application was applied using sources selected by farmers during the NMR interview from a list including diammonium phosphate, muriate of potash, single superphosphate, and four locally available NPK-containing mixed fertilizers. Urea was added early to fill any deficit in required N not meet by the farmer-selected sources.

Each NMR plot in kuruvai and samba had two embedded 25 m^2^ plots. One embedded plot referred to as NMR + K received an additional 40 kg K ha^−1^ as muriate of potash at panicle initiation. The other embedded plot, in an area designated at the start of each trial, received no additional fertilizer and served as an NMR reference treatment. All P and K rates are expressed on an elemental basis.

The BFR was 150 kg N ha^−1^, 22 kg P ha^−1^, and 42 kg K ha^−1^ as recommended by Tamil Nadu Agricultural University for irrigated inbred rice on all soil types in the Cauvery Delta, when a soil testbased fertilizer recommendation was not available (TNAU 2016). In BFR, all P was applied basal while fertilizer N and K were applied in four equal splits as 25% basal, 25% at active tillering, 25% at panicle initiation, and 25% at heading stage. All NMR and BFR plots received a basal application of 25 kg zinc sulfate ha^−1^ because soils in the study area are often low in extractable zinc. NMR did not have the ability to determine field-specific requirements for application of zinc.

The amount, method, and time for N, P, K, and zinc application in the FFP treatment were as determined through the interview of the farmer at the start of the season, and they were unique for each field trial. Online Resource 2—Part 2 reports the ranges in rates of fertilizer N, P, and K for FFP as compared to NMR. Researchers managed all fertilizer applications, including for FFP. All trials used transplanted rice, except three trials in samba using direct seeded rice. The farmer at each trial site uniformly managed land preparation, retention of residue from the previous crop, rice variety, crop establishment method (transplanting or direct seeding), and pest and disease control across the three treatments. Hence, the differences in crop performance and yield in a field trial reflect only differences in fertilizer management.

### Measurements and data analysis

The total cost of fertilizers included the cost of fertilizer sources plus the labor used for fertilizer applications. The costs of fertilizer sources used the prevailing local prices for farmers at the time of the research. The prices per 50 kg were 4.3 US$ for urea, 18.6 US$ for diammonium phosphate, 12.7 US$ for KCl, 5.9 US$ for single superphosphate, 14.3 US$ for 17–17–17 and 20–20–0 compound fertilizer, and 36.5 US$ for zinc sulfate. Labor for fertilizer application was estimated as 9.5 US$ ha^−1^ for each time of application. Other costs such as seed, labor (other than for fertilizer application), crop protection, and irrigation were not included in the financial analysis because they were similar for all the treatments in a given field trial. Grain yield was measured for three randomly selected 5 m^2^ areas in each plot and expressed at 14% water content.

Gross return was calculated as the product of grain yield and minimum support price for rice (US$ 222 per Mg of rice) at the time of the field research (DES 2015). Gross return above fertilizer cost was calculated as the difference between gross return and the total cost of fertilizer (Dobermann et al. 2004a). Added net benefit for NMR relative to FFP and BFR was calculated as the difference in gross return above fertilizer cost between NMR and the other treatment. Financial analyses are reported in US$ using an exchange rate of 1 US$ = 63 Indian rupees (INR). Partial factor productivity of added N (PFP) expressed in kg grain per kg N was calculated as follows

PFP = 1000 × GY/FN

where FN is fertilizer N expressed in kg ha^−1^ and GY is grain yield expressed in Mg ha^−1^ (Dobermann et al. 2004a).

Data were subjected to analysis of variance (ANOVA) using R version 3.5.1. For each growing season, ANOVA mixed models were fitted for the rate of fertilizer (N, P, and K), yield, cost of fertilizer, PFP, gross return above fertilizer cost, and added net benefit in which treatment was used as a fixed effect and farmer was a random effect. Separation of means used Duncan’s new multiple range test at *P* ≤ 0.05.

## Results

### Fertilizer use and grain yield

NMR used less fertilizer than BFR (Table 1). Rates of fertilizer N and P were significantly lower (*P* ≤ 0.05) for NMR than BFR in all three seasons, and rates of fertilizer K were significantly lower for NMR than BFR in two of the three seasons (kuruvai and thaladi). Rates of fertilizer K were high with NMR in samba because NMR estimated lower inputs of K from irrigation water in samba than kuruvai and lower inputs of K from crop residue in samba than thaladi (Online Resource 1—Part 2). The labor cost for fertilizer application averaged 9.5 US$ ha^−1^ less for NMR than BFR because of one less time of application. Lower use of fertilizer and labor with NMR resulted in significantly lower total fertilizer cost for NMR than BFR (Table 2). Zinc fertilization was identical for NMR and BFR and did not affect relative fertilizer costs.

**Table 1:** Amounts of fertilizer N, P and K applied with fieldspecific nutrient management through Nutrient Manager for Rice (NMR), a uniform fertilizer application using the blanket fertilizer recommendation (BFR), and farmer’s fertilizer practice (FFP) in three rice-growing seasons in Tamil Nadu, India

Nutrient	Treatment	Rate (kg ha^−1^)		
		Kuruvai	Samba	Thaladi
N	NMR	120b	130b	130b
	BFR	150a	150a	150a
	FFP	117b	117c	132b
P	NMR	14b	15b	13b
	BFR	22a	22a	22a
	FFP	21a	20a	22a
K	NMR	30b	52a	31b
	BFR	42a	42b	42a
	FFP	44a	53a	40a

Means within a column for a nutrient followed by the same letter are not different at *P* ≤ 0.05

Zinc sulfate was applied at 25 kg ha^−1^ with NMR and BFR in all trials. Zinc sulfate was applied with FFP in 15% of the trial in samba and 20% of the trials in thaladi

**Table 2:** Total fertilizer cost, rice grain yield, partial factor productivity of added N (PFP), gross return above fertilizer cost, and added net benefit for field-specific nutrient management through Nutrient Manager for Rice (NMR), a uniform fertilizer application using the blanket fertilizer recommendation (BFR), and farmer’s fertilizer practice (FFP) in three ricegrowing seasons in Tamil Nadu, India

Parameter	Treatment	Kuruvai	Samba	Thaladi
Total fertilizer cost (US$ ha^−1^)	NMR	111c	129b	111c
	BFR	142a	142a	142a
	FFP	117b	122b	121b
Grain yield (Mg ha^−1^)	NMR	5.3a	5.3a	3.9a
	BFR	5.1ab	5.3a	3.9a
	FFP	4.6b	4.7b	3.6a
PFP(kg grain kg^−1^ N)	NMR	44a	41a	30a
	BFR	34b	35b	26a
	FFP	41a	43a	29a
Gross return above fertilizer cost (US$ ha^−1^)	NMR	1055a	1051a	749a
	BFR	993ab	1035a	720a
	FFP	906b	916b	680a
Added net benefit (US$ ha^−1^)	NMR-BFR	62a	16b	29a
	NMR-FFP	149a	134a	70a

Means within a column for a parameter followed by the same letter are not different at *P* ≤ 0.05

NMR did not reduce rates of fertilizer N compared to FFP, but rates of fertilizer P were lower for NMR than FFP in all three seasons, and rates of fertilizer K were lower with NMR than FFP in two of the three seasons (Table 1). Less use of fertilizer P with NMR than FFP helped reduce cost of fertilizer N with NMR compared to FFP because a larger fraction of the total fertilizer N with NMR was from urea rather than costlier NP- and NPK-containing fertilizer sources. Use of zinc fertilizer was more for NMR than FFP because none of the 14 farmers in kuruvai applied zinc, only 15% of farmers in samba applied zinc, and only 20% farmers in thaladi applied zinc. Labor use was less with NMR than FFP in 68% of the trials because of one less time for fertilizer application (i.e., top dressing) with NMR. The net effect of differences in fertilizer use was lower total fertilizer cost with NMR than FFP in two of the three seasons (Table 2).

Historical yields reported by farmers during NMR interviews averaged 4.8 Mg ha^−1^ in kuruvai and samba and 4.4 Mg ha^−1^ in thaladi, but the range in reported yields was large (Table 3). NMR target yields were set higher than reported historical yields in each season (Table 3). Target yield for the entire 74 field trials ranged up to 2.5 Mg ha^−1^ higher than reported historical yield and was on average 1.0 Mg ha^−1^ higher than the reported historical yield (Online Resource 1—Part 1). Standard deviations and ranges were smaller for target yield than reported historical yield (Table 3) because target yields were set within a relatively narrow range determined by a baseline yield for the selected rice variety (Online Resource 1—Part 1).

**Table 3:** Historical yield reported by farmers, target yield with field-specific nutrient management through Nutrient Manager for Rice (NMR) recommendations, and the measured yield attained with NMR, expressed as a percentage of the target yield, in three rice-growing seasons in Tamil Nadu, India

Parameter	Kuruvai	Samba	Thaladi
Reported historical yield (Mg ha^−1^)			
Mean	4.8	4.8	4.4
Standard deviation	0.8	0.8	0.6
Range	3.6–6.1	3.6–6.4	3.6–5.6
NMR target yield (Mg ha^−1^)			
Mean	5.4	5.8	5.8
Standard deviation	0.3	0.4	0.3
Range	4.6–6.1	5.5–6.9	5.2–6.2
NMR measured yield (% of target yield)			
Mean	98	91	67
Standard deviation	10	12	14
Range	78–113	57–114	47–100

NMR target yield was relatively low in kuruvai (Table 3) because rice was transplanted after 15 June in all 14 trials due to late availability of irrigation water from late release of canal water. High yields can be achieved with sufficient irrigation water in kuruvai because of favorable solar radiation, but yields tend to decrease when crop establishment is delayed (Suganthi et al. 2003). NMR consequently reduced target yield when rice was transplanted after 15 June (Online Resource 1—Part 1). NMR target yield was relatively high in thaladi because high-yielding rice varieties were selected by most farmers, and NMR considered a baseline yield that can be attained by a variety when setting target yield (Online Resource 1—Part 1). NMR did not reduce target yield in thaladi, as in kuruvai, for delayed crop establishment.

**Fig.1:**
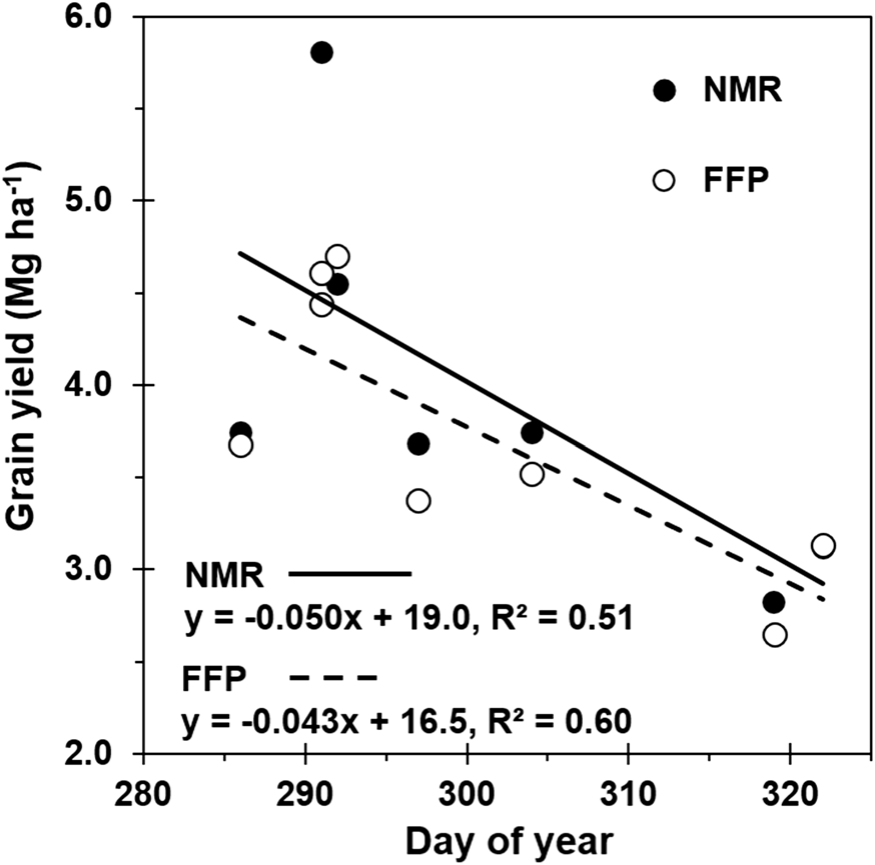
Effect of sowing date on rice grain yield for field- specific nutrient management through Nutrient Manager for Rice (NMR) and farmer’s fertilizer practice (FFP) in the thaladi season in Tamil Nadu, India

In kuruvai the mean yield measured with NMR (5.3 Mg ha^−1^ , Table 2) approximated the target yield (measured yield = 98% of target yield) (Table 3). In samba the yield with NMR (5.3 Mg ha^−1^) averaged 91% of the target yield. In thaladi the NMR recom-mendation failed to achieve the target yield (measured yield of 3.9 Mg ha^−1^ = 67% of target yield). Delayed crop establishment in kuruvai resulted in relatively late establishment of the subsequent thaladi crop. The most common variety in thaladi was ADT 38, which was grown on light-textured soil in 8 of the 20 trials with date for sowing seed to produce rice seedlings ranging from 13 Oct to 18 Nov. Yield with NMR and FFP decreased with sowing date (Fig. 1). Yield for sowing before 20 Oct (day of year = 293) averaged 4.7 Mg ha^−1^ with NMR and 4.4 Mg ha^−1^ with FFP. Mean yield for sowing after 20 Oct was markedly lower 3.3 Mg ha^−1^ with NMR and 3.2 Mg ha^−1^ with FFP. The effect of crop establishment date on rice yield was not considered by NMR when setting target yield for thaladi.

Measured grain yield with NMR did not signifi-cantly exceed yield with BFR (Table 2), but PFP of added N was significantly higher for NMR than BFR in two of the three seasons (Table 2) because NMR achieved comparable yields as BFR with less use of fertilizer N (Table 1). NMR increased grain yield compared to FFP in two of the three seasons (Table 2), but PFP of added N was not higher for NMR than FFP (Table 2) because NMR used the same or more fertilizer N than FFP (Table 1).

### Financial analysis

NMR did not significantly increase gross return above fertilizer cost compared to BFR, but gross return above fertilizer cost was significantly greater for NMR than FFP in two of the three seasons (Table 2). Higher yields for NMR than FFP resulted in the higher gross return above fertilizer cost for NMR than FFP in kuruvai and samba. High gross return above fertilizer cost for NMR resulted in positive mean added net benefit with NMR. Added net benefit arising from the use of NMR rather than FFP (NMR-FFP in Table 2) averaged 149 US$ ha^−1^ in kuruvai, 134 US$ ha^−1^ in samba, and 70 US$ ha^−1^ in thaladi. Use of NMR rather than BFR (NMR-BFR in Table 2) resulted in smaller added net benefits averaging 62 US$ ha^−1^ in kuruvai, 16 US$ ha^−1^ in samba, and 29 US$ ha^−1^ in thaladi.

**Fig. 2:**
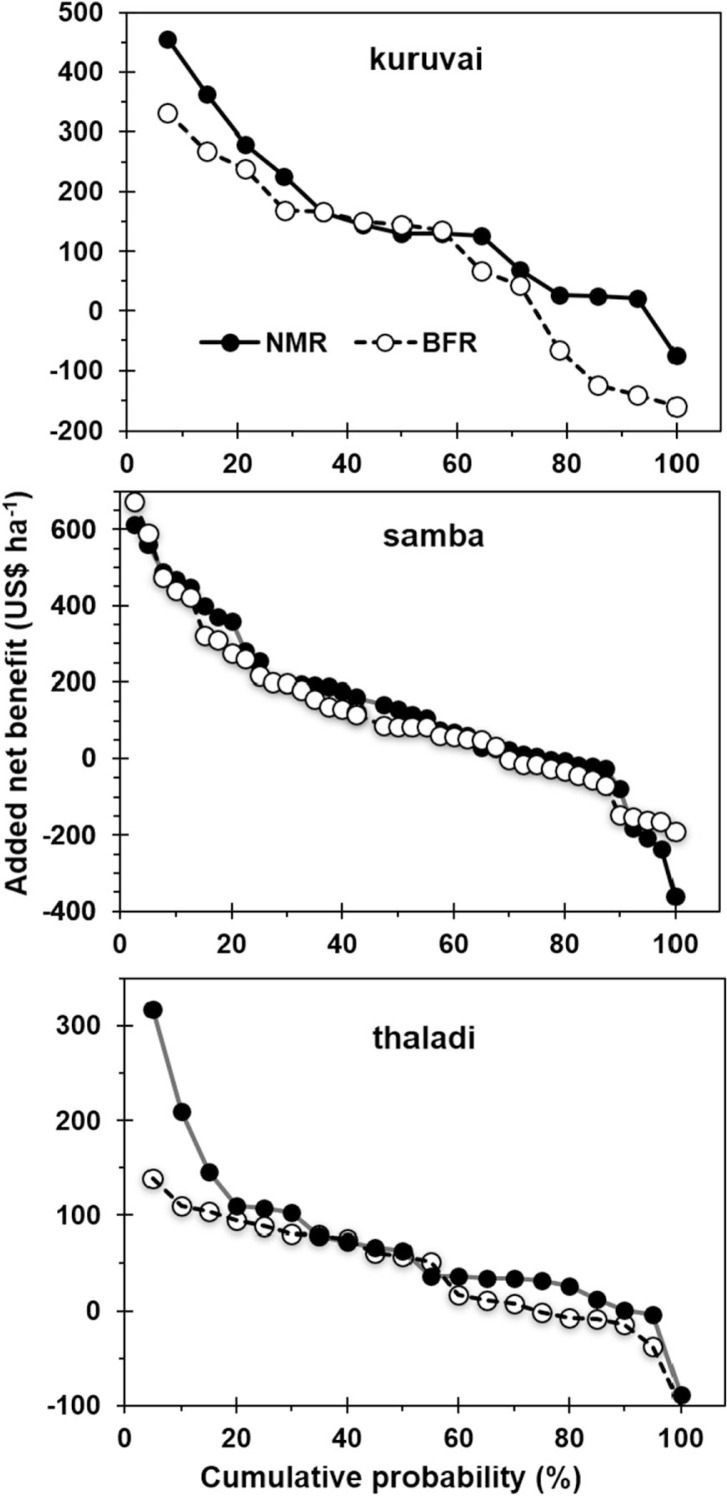
Cumulative probability of exceeding an added net benefit by switching from the farmer’s fertilizer practice (FFP) to either field-specific nutrient management through Nutrient Manager for Rice (NMR) or a uniform fertilizer application using the blanket fertilizer recommendation (BFR) in three ricegrowing seasons in the Cauvery Delta in Tamil Nadu, India

An advantage of NMR compared to BFR was reduced risk of financial loss. The probability of exceeding a threshold added net benefit was higher with a switch from FFP to NMR than to BFR (Fig. 2). For example, with a switch from FFP to NMR the probability of obtaining ≥ 25 US$ ha^−1^ added net benefit was approximately 86% in kuruvai, 69% in samba, and 81% in thaladi. By comparison, the probability with a switch from FFP to BFR was less: approximately 72% in kuruvai, 68% in samba, and 59% in thaladi. The likelihood of financial loss (i.e., negative added net benefit) was consistently lower with a switch from FFP to NMR than to BFR. The likelihood with a switch from FFP to NMR averaged 18% across all trials (7% in kuruvai, 25% in samba, and 10% in thaladi). The likelihood of financial loss, by comparison, with a switch from FFP to BFR averaged 31% (29% in kuruvai, 33% in samba, and 30% in thaladi).

**Fig. 3:**
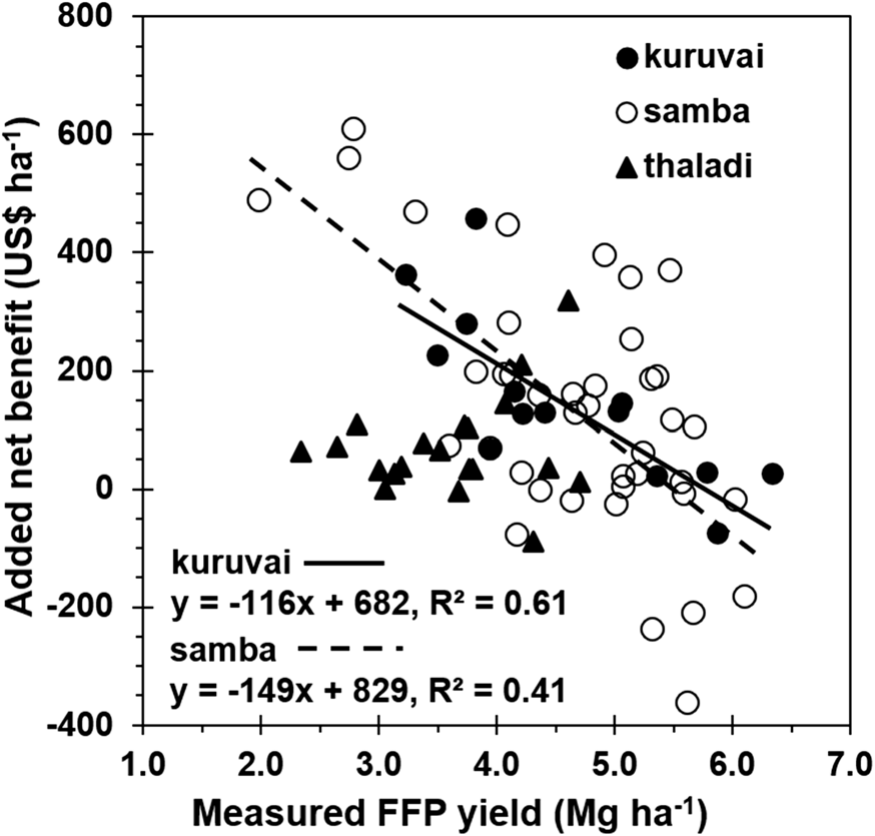
Comparison of actual yields measured for farmer’s fertilizer practice (FFP) and added net benefit for Nutrient Manager for Rice (NMR) relative to FFP in three rice-growing seasons in the Cauvery Delta in Tamil Nadu, India

Lower benefit from NMR than FFP (i.e., negative added net benefit) was not associated with a particular rice variety or higher total fertilizer cost for NMR than FFP. Added net benefit for NMR relative to FFP or BFR was not correlated with NMR target yield; but added net benefit for NMR relative to FFP was inversely related to yield with FFP in kuruvai and samba (Fig. 3). Added net benefit tended to decrease with increasing FFP yield, and mean yield in the 13 of 74 trials with negative added net benefit was lower for NMR (4.7 Mg ha^−1^) than FFP (5.1 Mg ha^−1^). Added net benefit for NMR relative to FFP or BFR was, however, not correlated with reported historical yield in the three seasons. Farmers in our study most likely to benefit from NMR could not be identified from historical yields reported during NMR interviews before crop establishment.

### Nutrient balances

NMR targeted full maintenance of soil P by setting P rate equal to estimated crop removal of P (Online Resource 1—Part 2). The achievement of target yield would consequently result in estimated P balance = 0 (input from fertilizer = output by crop removal). The slightly positive P balance for NMR in thaladi (Table 4) reflects the failure to achieve the target yield (Table 3). Estimated P balances for BFR and FFP were positive in each season indicating mean P rates exceeded the minimum required to prevent mining of soil P (Table 4). Rates of fertilizer P varied more for farmer’s practice (FFP) than NMR (Online Resource 2—Part 2) resulting in high standard deviations with P balances for FFP (Table 4). Despite positive mean P balances for FFP, 7% of the farmers in kuruvai and 20% of farmers in samba had P balances more negative than - 3 kg ha^−1^ due to low application of P.

**Table 4:** Estimated P and K balances—based on nutrient inputs from fertilizer, crop residues, and irrigation water—for field-specific nutrient management through Nutrient Manager for Rice (NMR), a uniform fertilizer application using the blanket fertilizer recommendation (BFR), and farmer’s fertilizer practice (FFP) in three rice-growing seasons in Tamil Nadu, India

Treatment	Estimated P balance (kg P ha^−1^)			Estimated K balance (kg K ha^−1^)		
	Kuruvai	Samba	Thaladi	Kuruvai	Samba	Thaladi
NMR	0 (1.4)	1 (2.0)	5 (2.2)	− 28 (11.1)	− 24 (13.1)	26 (14.1)
BFR	9 (1.2)	8 (2.5)	14 (1.8)	− 5 (6.9)	− 28 (14.4)	36 (15.9)
FFP	9 (7.0)	8 (10.8)	15 (7.3)	5 (18.7)	− 7 (32.2)	41 (25.5)
NMR target	0 (0.3)	0 (0.2)	0 (0.2)	− 30 (7.0)	− 33 (5.5)	− 4 (6.8)

Values in parenthesis are standard deviations

Nutrient balances assume K input from irrigation water of 25 kg ha^−1^ in kuruvai, 8 kg ha^−1^ in samba, and 4 kg ha^−1^ in thaladi; no input of P from irrigation water; no input of P and K from organic materials other than rice residue; and no output of P and K by processes other than crop removal

NMR targeted partial maintenance of soil K by allowing some drawdown of soil K after estimating K output by crop removal and K input from irrigation water (Online Resource 1—Part 2, Table 4). Whereas inputs of P with irrigation water are typically negligible, inputs of K with irrigation water can be appreciable (Witt and Dobermann 2004). NMR used reported mean K concentration of 2.6 mg L^−1^ for irrigation water from tube wells in the Cauvery Delta (Buresh et al. 2010) to estimate K inputs from irrigation water as 25 kg ha^−1^ in kuruvai, 8 kg ha^−1^ in samba, and 4 kg ha^−1^ in thaladi (Online Resource 1—Part 2). Field trials in kuruvai and samba did not receive crop residues from the previous rice crop resulting in removal of large amounts of K. The K balances were consequently negative (K input from fertilizer and irrigation water < K output by crop removal) for all treatments except FFP in kuruvai (Table 4). Field trials in thaladi on the other hand received crop residue from the previous kuruvai crop, which was combine harvested, resulting in removal of much less K. The K balance for NMR was positive in thaladi (Table 4) because NMR failed to achieve the target yield (Table 3). Rates of fertilizer K varied more for farmer’s practice (FFP) than NMR (Online Resource 2—Part 2) resulting in high standard deviations with K balances for FFP (Table 4). As a result, K balances in samba were more negative with FFP than NMR for 30% of the trials despite less negative mean K balance with FFP than NMR.

The failure of NMR to prevent mining of soil K in kuruvai and samba can raise concern whether fertilizer K was sufficient to prevent K deficiency. Application of an additional 40 kg K ha^−1^ to NMR plots did not increase yield in these two seasons (Table 5) indicating K rates with NMR were sufficient to overcome K deficiencies. It, however, cannot be determined from our short-term study whether rice yields could be sustained with long-term use of K rates targeting less than full maintenance of soil K.

**Table 5:** Effect of an additional 40 kg K ha^−1^ on rice grain yield with field-specific nutrient management through Nutrient Manager for Rice (NMR) in two rice-growing seasons in Tamil Nadu, India

Treatment	Grain yield (Mg ha^−1^)	
	Kuruvai	Samba
NMR	5.2a	5.4a
NMR + K	5.1a	5.2a

Means within a column for a parameter followed by the same letter are not different at *P* ≤ 0.05

## Discussion

### Performance of NMR

Our study showed the capability of NMR to lower total fertilizer cost and increase PFP of added N compared to BFR, even though yield was not significantly higher for NMR than BFR (Table 2). BFR supplied excessive amounts of N and P fertilizer as indicated by low PFP of added N (Table 2) and P rates in excess for maintenance of soil P (Table 4). Despite the superior performance of NMR, a financial loss for 18% of the farmers using NMR suggested that efforts to further improve NMR are warranted. Farmers not benefitting from NMR could not be distinguished from other farmers in our study based on differences in total fertilizer cost, reported historical yield, NMR target yield, soil texture, past use of composted farmyard manure, and past exposure to researcher-managed field demonstrations. The inverse relationship between added net benefit and farmer’s measured yield (Fig. 3) suggested dissemination of NMR for highest impact could target relatively lower yielding farmers and future research could examine the feasibility of further improving nutrient management and NMR for high-yielding farmers.

The mean PFP of added N for NMR in our study (30–44 kg kg^−1^) (Table 2) was lower than the mean of 51 kg kg^−1^ reported for SSNM in the Cauvery Delta by Nagarajan et al. (2004) due to lower yields in our study. Mean yield with SSNM was approximately 6.0 Mg ha^−1^ across kuruvai and thaladi in 1997–2000 (Nagarajan et al. 2004) as compared to 5.3 Mg ha^−1^ in kuruvai and 3.9 Mg ha^−1^ in thaladi for NMR in our study with different farmers in 2014–2015. Although some differences in yield between Nagarajan et al. (2004) and our study might be due to differences in farmers and rice fields, the relatively low yield in our study was likely associated at least partly with late crop establishment. Solar radiation and yield decrease with a delay in crop establishment between May and July in kuruvai (Suganthi et al. 2003). The kuruvai crop in our study was sown relatively late on 8-23 June and then transplanted in July due to late availability of irrigation water. NMR reduced target yield and accompanying fertilizer rates when transplanting occurred after 15 June (Online Resource 1— Part 1). This likely contributed to the good fit of measured NMR yield with NMR target yield in kuruvai (Table 3).

Late transplanting in kuruvai resulted in a corre-sponding delay for the subsequent thaladi crop. Thaladi in our study was sown from 8 Oct to 18 Nov and harvested in February and March, which was about one month later than typical during earlier SSNM research in 1997–2000 (Dobermann et al. 2004a) and corresponded to a period of lower solar radiation (Timsina et al. 2011). Low solar radiation and shift of the cropping period beyond the rainy season likely contributed to the relatively low yield in thaladi (Table 2) and the decrease in yield with date of sowing in thaladi (Fig. 1). Samba was also relatively late in our study with crop establishment from 28 Aug–30 Sep and harvest in January and February.

NMR target yield in thaladi, and to a lesser extent in samba, overestimated yield achievable with NMR (Table 3) resulting in excessive application of fertilizer. NMR did not adjust target yield in thaladi or samba, unlike in kuruvai, for date of crop establishment. Our findings (Fig. 1) suggest a probable improvement to NMR would be to reduce target yield in thaladi and samba when the crop is established later than a threshold date.

Higher yield for NMR than FFP in kuruvai without higher rates of fertilizer N, P, and K might be attributed to improved distribution of fertilizer N for NMR to better match the need of the crop for supplemental N. NMR and BFR in all trials received a blanket application zinc to ensure the comparison of NMR with BFR was not affected by zinc. Most farmers on the other hand did not apply zinc. FFP included application of zinc in only 14% of the trials. Higher yield for NMR than FFP might then also be associated with zinc; and the relative benefits of NMR from improved distribution of fertilizer N versus application of zinc cannot be separated in our study.

The partial factor productivity of fertilizer N (PFP) was never lower for NMR than either BFR or FFP (Table 2). This suggested that the number of fertilizer N applications could safely be reduced from four used in all BFR trials and 69% of the FFP trials to three used for NMR. This reduction in number of applications would reduce labor cost for application of fertilizer.

Lower PFP in our study than in earlier SSNM research in the Cauvery Delta and failure of higher rates of N for BFR to achieve higher yield than NMR (Table 2) suggested that yield with NMR was not limited by insufficient N. There consequently might be scope to reduce the rate of fertilizer N calculated by NMR to achieve a target yield. Fertilizer N rate could be reduced by targeting a higher agronomic efficiency in the calculation of fertilizer N rate than 14–15 kg kg^−1^ currently used in NMR (Online Resource 1—Part 2). Agronomic efficiencies of fertilizer N > 15 kg kg^−1^ are achievable for irrigated, high-yielding rice when using good crop management practices (Peng et al. 1996).

The scope for further improvement of fertilizer P and K calculations in NMR appears to be limited because further reductions in P rates with NMR would risk mining of soil P and further reductions in K rates with NMR would risk even more mining of soil K (Table 4). The P and K rates in NMR were already lower than P and K rates determined with an earlier version of SSNM evaluated in the Cauvery Delta during 1997–2004 by Nagarajan et al. (2004) and Rajendran et al. (2010). Lower P and K rates for NMR likely contributed to the higher added net benefits reported for NMR relative to FFP in our study (70–149 US$ ha^−1^) than reported for SSNM relative to FFP by Nagarajan et al. (2004) (56-85 US$ ha^−1^) and Rajendran et al. (2010) (49–95 US$ ha^−1^).

### Lessons for widescale improvement of P and K management for rice

Our study highlighted an immediate opportunity to lower fertilizer costs for an existing recommendation (BFR) by using an SSNM-based full nutrient balance approach in NMR to set P rates (Online Resource 1— Part 2). Use of a nutrient balance to calculate P required to achieve a target yield would also help avoid under and over application of fertilizer P, which was common for farmers in our study (Online Resource 2—Part 2). The nutrient balance approach merits widescale consideration for determining sustainable fertilizer P requirements for rice.

The determination of fertilizer requirements is more complex for K than for P because K balances are more influenced by management of crop residues and nutrient input from irrigation water (Buresh et al. 2010). Rice residues contain 80–85% of total aboveground plant K but only about 30% of total aboveground plant P (Dobermann and Fairhurst 2000). A full nutrient balance approach with complete removal of rice residue, as is common across South Asia, would recommend relatively high K rates to avoid mining of soil K. The partial nutrient balance approach allows some mining of soil K to reduce fertilizer cost and achieve higher short-term net income.

NMR determined fertilizer K rates using a partial nutrient balance approach (Buresh et al. 2010; Witt and Dobermann 2004), which allowed mining of soil K (Online Resource 1—Part 2). Negative K balances were targeted with NMR in each season, but the actual K balance with NMR depended on the nearness of the achieved yield to the target yield (Table 4). The absence of higher yield with NMR ? K than NMR (Table 5) confirmed NMR used sufficient K rates to avoid short-term K deficiency. The partial nutrient balance approach provided an improvement to a blanket recommendation for K by enabling a relatively straightforward adjustment of K rate for target yield and management of crop residue. The partial nutrient balance approach merits consideration for determining fertilizer K requirements for rice especially when achievable yields and management of crop residues vary among farmers and seasons.

More negative K balances with NMR in kuruvai than the succeeding thaladi season (Table 4) sug-gested scope for improving NMR by redistributing some fertilizer K from thaladi to kuruvai in this production system with two irrigated rice crops per year. One improvement could be allowing comparable drawdown of soil K reserves for thaladi and kuruvai in the calculation of K rates, rather than the currently greater drawdown in kuruvai than thaladi (Online Resource 1—Part 2). Another improvement could be adjustment in the estimated input of K from irrigation water to better account for source of irrigation water (i.e., well or canal), estimated use of irrigation water in a season, and estimated concentration of K in irrigation water. Application of K from other organic sources, including composed farmyard manure, might warrant consideration in the estimation of K rates.

Fertilizer rates determined with NMR were based on target yields (Buresh et al. 2010), which were set higher than historical yield reported by the farmer (Table 3). NMR additionally used a baseline yield for each rice variety, as obtained from local rice experts and past field trials, to set target yield (Online Resource 1—Part 1). An alternative to such a baseline yield for a variety could be to estimate potential yield for a selected variety and crop establishment date using historical climate and crop simulation models. Potential yield could then be further adjusted to estimate a field-specific target yield, by usi ng factors such as forecasted supply of irrigation water, anticipated crop management practices, and seasonal weather forecasts.

## Conclusions

Precise field-specific fertilizer management on small landholdings with NMR rather than uniform application of fertilizer across a wide production area could for the conditions in our study reduce total fertilizer cost, increase partial factor productivity of added N, and reduce the likelihood of financial loss for an individual farmer. The capability of NMR to adjust fertilizer rates for a target yield set for each small landholding contributed to reducing fertilizer costs and reducing the risk of financial loss when the recommended fertilizer practice was used by a farmer. The calculation by NMR of field-specific fertilizer N, P, and K requirements using SSNM principles depends heavily on the estimation of a target yield, which can be reliably achieved by a farmer. Our findings suggest the date of crop establishment warrants consideration in the estimation of target yield, especially when the date of rice sowing or transplanting varies among nearby farmers.
