# Supplementary material for: Web-based tool for calculating field-specific nutrient management for rice in India
Source: Nutr Cycl Agroecosyst. 2018 Oct 22;113:21–33. doi: 10.1007/s10705-018-9959-x (PMC7357723; doi:10.1007/s10705-018-9959-x)
Supplement: Supplementary file 1 [file NCA-2019-s10705-018-9959-x-S1.pdf]

Online Resource 1. Operation of Nutrient Manager for Rice (NMR) for the Cauvery Delta, Tamil Nadu, India

Article title:
Web-based tool for calculating field-specific nutrient management for rice in India

Journal name:
Nutrient Cycling in Agroecosystems

Author names:
Sheetal Sharma, P. Panneerselvum, Rowena Castillo, Shriram Manohar, Raj Rajendran, V. Ravi, Roland J. Buresh

Author affiliations:
Sheetal Sharma, P. Panneerselvum, Shriram Manohar
International Rice Research Institute, India

Rowena Castillo, Roland J. Buresh
International Rice Research Institute, DAPO Box 7777, Metro Manila, Philippines

Raj Rajendran
Coconut Research Station, Tamil Nadu Agricultural University, Veppankulam, Thanjavour, Tamil Nadu, India

V. Ravi
Tamil Nadu Rice Research Institute, Tamil Nadu Agricultural University, Aduthurai, Thanjavour, Tamil Nadu, India

Email address of corresponding author:
Sheetal Sharma: Sheetal.sharma@irri.org

Content

Part 1. NMR calculation of target yield 2
Part 2. NMR calculation of fertilizer N, P, and K rates 5
Part 3. NMR calculation of dates for application of fertilizer N 7
Part 4. Information included within NMR recommendations 10

Part 1. NMR calculation of target yield

Nutrient Manager for Rice (NMR) uses information on the rice variety selected by the farmer, yield reported by the farmer for previous years, and sowing date to calculate a target yield, which is then used to determine the amounts of required fertilizer N, P, and K. Extension workers and researchers with a mobile device or computer can use NMR to interview a rice farmer before crop establishment using interactive questions about a specific rice field. During this interview the farmer indicates for the upcoming season the rice variety, expected sowing date, and yield obtained in previous years (i.e., reported historical yield). After the interview of the farmer, NMR transmits collected information via the internet to a cloud-based server where it is stored and used to calculate target yield and fertilizer requirements. The cloud-based server also has a database containing a baseline yield for each rice variety commonly grown in the Cauvery Delta (Supplementary Table 1.1). The baseline yield for a rice variety was compiled and updated by local research and extension experts using results from field trials and experiences of rice farmers.

NMR sets a target yield for the rice variety selected by the farmer within a range between the baseline yield in the database (Supplementary Table 1.1) and an upper yield limit, which is 12% higher than the baseline yield. The target yield is normally set higher than the historical yield reported by a farmer (Supplementary Fig. 1.1) because improved management of fertilizer with NMR is assumed to increase yield of a farmer.

Target yield for the 74 field trials was on average 1.0 Mg ha^{-1} higher than the reported historical yield. The increase in target yield compared to reported historical yield was greatest when farmers reported relatively low yield for past years. Target yield was on average 1.6 Mg ha^{-1} higher than the reported historical yield for 38 field trials with reported historical yields <4.5 Mg ha^{-1}. Target yield was set lower than reported historical yield in only 3 of 74 trials (Supplementary Fig. 1.1) because of delayed transplanting. NMR automatically reduces the typical yield of a variety (Supplementary Table 1.1) by 10%, and correspondingly reduces the target yield, when rice in kuruvai is transplanted after 15 June.

NMR aims to increase net income for the farmer by achieving higher yield with more efficient use of fertilizer. NMR does not set target yield above the upper yield limit, regardless of the reported historical yield, to minimize risk to the farmer of financial loss arising from increased cost for fertilizer without a commensurate increase in rice production. NMR does not use historical climate to set target yield because rice production and yield are strongly dependent on supply of canal irrigation water, which is typically independent of climate during the growing season. NMR assumes a farmer obtaining an NMR recommendation has assurance of a supply of irrigation water in the upcoming season.

Supplementary Table 1.1 Rice varieties with baseline yield and growth duration used by Nutrient Manager for Rice (NMR)

Rice variety	Baseline yield ($Mg\ ha^{-1}$)[a]	Growth duration (days)[b]
ADT (R) 45	6.1	101–110
ADT (R) 46	6.2	131–140
ADT (R) 47	6.2	111–120
ADT (R) 48	4.8	91–100
ADT 36	5.1	101–110
ADT 37	6.2	101–110
ADT 38	5.8	131–140
ADT 39	5.6	121–130
ADT 42	6.0	111–120
ADT 43	5.9	101–110
ADT 44	6.2	141–150
ADT 49	6.2	131–140
ADT 50	5.9	141–150
ASD 16	6.0	111–120
ASD 19	5.8	131–140
BPT 5204	5.5	131–140
CO (R) 50	6.5	131–140
CO 43	6.5	131–140
CO 51	5.2	101–110
CORH 2	6.1	131–140
CORH 3	6.4	111–120
CORH 4	7.3	131–140
CR 1009	6.5	151–160
IR 20	5.0	131–140
IR 50	6.5	101–110
Swarna Sub 1	5.5	131–140
TKM 9	5.5	101–110
TRY 1	5.2	131–140
TRY 3	6.0	131–140
White Ponni	4.5	131–140

[a]Baseline yield in kuruvai is reduced by 10% when rice is transplanted after 15 June
[b]The reported growth duration is for transplanted rice. Duration is reduced by ten days for direct seeded rice.

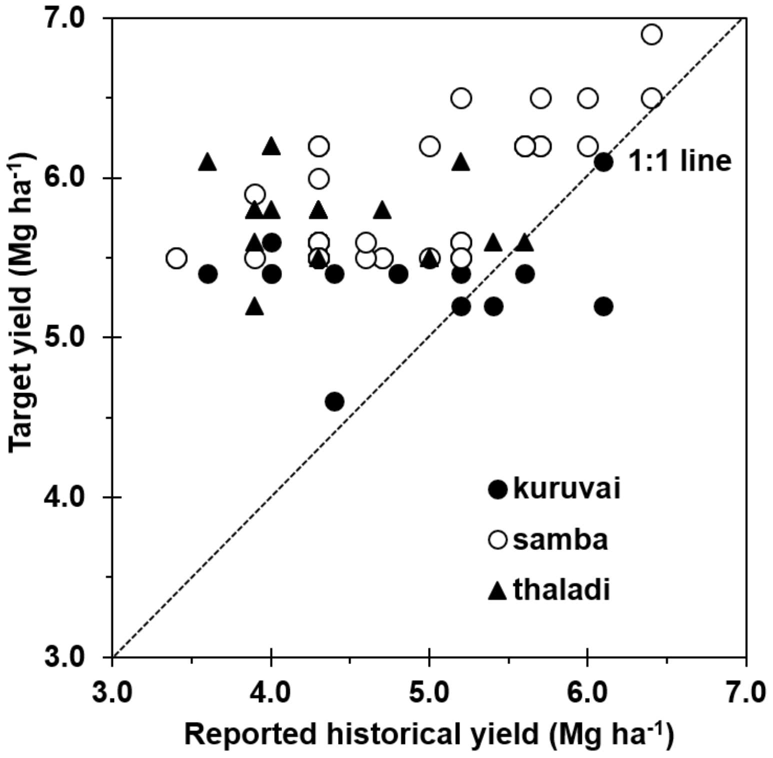

Supplementary Fig. 1.1 Comparison of historical yield reported for a season by farmers and target yield set by Nutrient Manager for Rice (NMR) for 74 field trials in three growing seasons in the Cauvery Delta in Tamil Nadu, India. Some data points in the figure represent more than one field trial.

Part 2. NMR calculation of fertilizer N, P, and K rates

NMR calculates field-specific fertilizer P and K rates using nutrient input-output balances for the field (Buresh et al. 2010):

$FP = GY_T \times RIE_P - P_{CR} - P_S$

$FK = GY_T \times RIE_K - K_{CR} - K_W - K_S$

where FP is rate of fertilizer P and FK is rate of fertilizer K with both expressed in kg ha^{-1}, GY_T is target grain yield expressed in Mg ha^{-1}, RIE_P is reciprocal internal efficiency for P and RIE_K is reciprocal internal efficiency for K with both expressed in kg of P or K in above-ground dry matter per 1 Mg of grain yield, P_{CR} is input of P in retained above-ground crop residues and K_{CR} in input of K in retained above-ground crop residues with both expressed in kg ha^{-1}, K_W is input of K with irrigation water for an entire cropping cycle expressed in kg ha^{-1}, and P_S and K_S are allowable drawdowns in soil P and K with both expressed in kg ha^{-1}.

RIE_P and RIE_K are set to 2.7 kg P and 15.9 kg K per Mg grain yield, respectively. These values were estimated by Buresh et al. (2010) for mature rice using regression analysis with data from plot-level measurements across Asia. The P and K inputs from residues of the previous rice crop (P_{CR} and K_{CR}) are determined from the quantity and nutrient content of above-ground biomass retained in the field after harvest of the previous rice crop (Buresh et al. 2010):

$P_{CR} = GY \times RIE_P \times (1 - HI_P) \times CRR$

$K_{CR} = GY \times RIE_K \times (1 - HI_K) \times CRR$

where GY is estimated grain yield of the previous rice crop expressed in Mg ha^{-1}, HI_P and HI_K are P and K harvest indices for rice expressed in kg nutrient in grain per kg nutrient in total above-ground dry matter, and CRR is the fraction of total residue from the previous rice crop retained in the field. An estimate of grain yield for the previous rice crop (GY) is obtained through the interview of the farmer using the interactive questions of NMR. NMR uses published data (Dobermann and Fairhurst 2000; Buresh et al. 2010) to set $HI_P = 0.7$ and $HI_K = 0.15$.

Residues from the rice crop before kuruvai and samba are normally removed from the field during the long fallow period between crops, regardless of the method of rice harvest (i.e., manual or combine). NMR consequently assumes residue from the rice crop before kuruvai and samba is largely removed from the field, and the fraction of total residue from the previous rice crop retained in the field (CRR) is set to only 0.15 for kuruvai and 0.1 for samba. Thaladi on the other hand typically receives some residue from the kuruvai crop, which is harvested either manually or with a combine immediately before thaladi. When used to make a recommendation for thaladi, NMR determines the method of harvest (manual or combine), cutting height for manual harvest, and management of residues after combine harvest for the previous kurvai crop through the interview of the farmer. CRR for thaladi is set to 0.2 for manual harvest with low cutting height, 0.4 for manual harvest with high cutting height, 0.6 for combine harvest with removal of straw, and 1.0 for combine harvest without removal of straw.

NMR sets K input from irrigation water (K_W) to 25 kg K ha^{-1} in kuruvai, 8 kg K ha^{-1} in samba, and 4 kg K ha^{-1} in thaladi. The input of K is assumed to be highest in kuruvai due to usage of large quantities of irrigation water from wells, which often contains higher concentration of K than surface water from irrigation canals. The use of irrigation water is typically less during samba and thaladi, which occur in the rainy season, and NMR assumes that much of the irrigation water in samba and thaladi originates from canals.

NMR aims for full maintenance of soil P by setting $Ps = 0$ to avoid a net drawdown in soil P. NMR on the other hand allows a drawdown in soil K. Soils contain varying amounts of K-bearing minerals, which supply indigenous K to crops (Bijay-Singh et al. 2004). The magnitude, temporal dynamics, and longevity of this supply of K can vary among soils (Majumdar et al. 2017). The SSNM-based determination of fertilizer K requirements provides the option for a partial nutrient balance approach with drawdown of soil K reserves (K_S), adjusted for soil and environmental conditions (Buresh et al. 2010). NMR allows an annual drawdown of soil K up to but not exceeding 25 kg K ha^{-1} for sandy soil and 50 kg K ha^{-1} for clayey soil. For samba with only one rice crop per year, Ks is 25 kg K ha^{-1} for sandy soil and 50 kg K ha^{-1} for clayey soil. Ks for kuruvai is 17 kg K ha^{-1}

for sandy soil and 34 kg K ha^{-1} for clayey soil, and Ks for thaladi is 8 kg K ha^{-1} for sandy soil and 16 kg K ha^{-1} for clayey soil. NMR obtains information during the interview of farmers on quantity of added composted farmyard manure, but applications of composted farmyard manure are usually relatively small and infrequent. NMR consequently does not consider inputs of N, P, and K from farmyard manure in the calculation of fertilizer rates.

NMR calculates field-specific fertilizer N rates using a yield gain approach (Buresh 2015):

$$FN = 1000 \times (GY_T - GY_{0N})/AE_N$$

where FN is fertilizer N expressed in kg ha^{-1}, GY_{0N} is grain yield without added N expressed in Mg ha^{-1}, and AE_N is agronomic efficiency of added fertilizer N expressed in kg increase in grain yield per kg applied fertilizer N. NMR sets AE_N = 14 kg kg^{-1} when GY_T <7 Mg ha^{-1} and 15 kg kg^{-1} when GY_T ≥7 Mg ha^{-1}. These values correspond to an average AE_N of 14.6 kg kg^{-1} reported for SSNM in earlier research in the Cauvery Delta (Nagarajan et al. 2004).

Y_{0N} serves as an indicator of indigenous N supply from sources other than fertilizer. It was estimated as a function of GY_T using results from 252 nutrient omission plot technique trials conducted in the Cauvery Delta in 1997–2004. In these trials, GY_T for a given trial was determined in plots receiving sufficient fertilizer to eliminate N, P, and K deficiencies and GY_{0N} in the trial was determined in plots receiving no fertilizer N (Witt et al. 2007). Regression analysis for results from the 252 trials provided the following relationship, which was used in NMR to estimate GY_{0N} (or indigenous N supply) for a given target yield:

$$GY_{0N} = 0.61 \times GY_T + 0.4$$

References

Bijay-Singh, Yadvinder-Singh, Imas P, Jian-Chang X (2004) Potassium nutrition of the rice–wheat cropping system. Advances in Agronomy 81:203–259

Buresh RJ (2015) Nutrient and fertilizer management in rice systems with varying supply of water. In: Drechsel P, Heffer P, Magen H, Mikkelsen R, Wichelns D (ed), Managing water and fertilizer for sustainable agricultural intensification. International Fertilizer Industry Association (IFA), International Water Management Institute (IWMI), International Plant Nutrition Institute (IPNI), and International Potash Institute (IPI), Paris, France, pp 187–208. http://www.iwmi.cgiar.org/Publications/Books/PDF/managing_water_and_fertilizer_for_sustainable_agricultural_intensification.pdf

Buresh RJ, Pampolino MF, Witt C (2010) Field-specific potassium and phosphorus balances and fertilizer requirements for irrigated rice-based cropping systems. Plant Soil 335:35–64

Dobermann A, Fairhurst T (2000) Rice: Nutrient disorders and nutrient management. Potash & Phosphate Institute (PPI) and Potash & Phosphate Institute of Canada (PPIC), Singapore and International Rice Research Institute (IRRI), Los Baños, Philippines. http://books.irri.org/9810427425_content.pdf

Majumdar K, Sanyal SK, Singh, VK, Dutta S, Satyanarayana T, Dwivedi BS (2017) Potassium fertiliser management in Indian agriculture: Current trends and future needs. Indian Journal of Fertilisers 13:20–30

Nagarajan R, Ramanathan S, Muthukrishnan P, Stalin P, Ravi V, Babu M, Selvam S, Sivanantham M, Dobermann A, Witt C (2004) Site-specific nutrient management in irrigated rice systems of Tamil Nadu, India. In: Dobermann A, Witt C, Dawe D (ed) Increasing productivity of intensive rice systems through site-specific nutrient management. Science Publishers, Enfield, NH (USA) and International Rice Research Institute (IRRI), Los Baños, Philippines, pp 101–123

Witt C, Buresh RJ, Peng S, Balasubramanian V, Dobermann A (2007) Nutrient management. In: Fairhurst T, Witt C, Buresh R, Dobermann A (ed) Rice: A practical guide to nutrient management. International Rice Research Institute (IRRI), Los Baños, Philippines and International Plant Nutrition Institute (IPNI) and International Potash Institute (IPI), Singapore, pp 1–45. http://books.irri.org/97898179494_content.pdf

Part 3. NMR calculation of dates for application of fertilizer N

NMR distributes fertilizer N in three doses corresponding to critical growth stages of early vegetative growth, tillering, and panicle initiation (Peng and Cassman 1998; Witt et al. 2007). Panicle initiation is normally about 60 days before physiological maturity in the tropics. The duration of a rice crop in the field, and hence the number of days between crop establishment and panicle initiation, can vary greatly depending of the seed-to-seed duration of the rice variety, the method of crop establishment (i.e., transplanting or direct seeding), and the age of transplanted seedlings. NMR estimates the number of days between crop establishment and N application at panicle initiation using information on seed-to-seed duration of the rice variety (Supplementary Table 1.1) selected by the farmer and information obtained from the farmer during the NMR interview on the planned method of crop establishment (i.e., manual transplanting, mechanical transplanting, direct wet seeding, and direct dry seeding) and the expected age for transplanted seedlings. NMR then calculates a time internal for the early N application and the number of days after crop establishment for N application at tillering, which is about midway between the early application and panicle initiation. The time interval for the early N application expands as the number of days between crop establishment and panicle initiation increases. The interval for the early N application is reduced when N is applied with a P containing fertilizer, such as diammonium phosphate. This ensures fertilizer P is applied sufficiently near to the time of crop establishment. Supplementary Tables 3.1 and 3.2 show how NMR adjusts the times for N application.

References

Peng S, Cassman KG (1998) Upper threshholds of nitrogen uptake rates and associated nitrogen fertilizer efficiencies in irrigated rice. Agron J 90:178–185

Witt C, Buresh RJ, Peng S, Balasubramanian V, Dobermann A (2007) Nutrient management. In: Fairhurst T, Witt C, Buresh R, Dobermann A (ed) Rice: A practical guide to nutrient management. International Rice Research Institute (IRRI), Los Baños, Philippines and International Plant Nutrition Institute (IPNI) and International Potash Institute (IPI), Singapore, pp 1–45. http://books.irri.org/97898179494_content.pdf

Supplementary Table 3.1 Recommended times for three applications of fertilizer N for transplanted rice as affected by method of transplanting, age of transplanted seedlings, growth duration of rice variety, and fertilizer source for early application of N and P

Transplanting method	Seedling age (days)	Growth duration (days)	Days after transplanting					
			Compound NP fertilizer[a]			Separate sources of N and P[b]		
			Early	Tillering	Panicle initiation	Early	Tillering	Panicle initiation
Mechanical	15	91–100	0–5	13–17	24–28	0–5	13–17	24–28
	15	101–110	0–7	18–22	32–36	0–14	22–26	32–36
	15	111–120	0–7	22–26	40–44	0–14	26–30	40–44
	15	121–130	0–7	27–31	48–52	0–14	30–34	48–52
	15	131–140	0–7	32–36	58–62	0–14	35–39	58–62
	15	141–150	0–7	36–40	68–72	0–14	40–44	68–72
	15	151–160	0–7	42–46	78–82	0–14	45–49	78–82
Manual[c]	<25	91–100	0	8–12	19–23	0	8–12	19–23
	<25	101–110	0–7	16–20	28–32	0–7	16–20	28–32
	<25	111–120	0–7	20–24	34–38	0–14	24–28	34–38
	≥25	91–100	0	–[e]	11–15	0	–[e]	11–15
	≥25	101–110	0–5	12–16	21–25	0–5	12–16	21–25
	≥25	111–120	0–7	16–20	28–32	0–7	16–20	28–32
Manual[d]	<35	111–120	0–7	16–20	28–32	0–7	16–20	28–32
	<35	121–130	0–7	20–24	34–38	0–14	24–28	34–38
	<35	131–140	0–7	24–28	43–47	0–14	27–31	43–47
	<35	141–150	0–7	29–33	53–57	0–14	33–37	53–57
	<35	151–160	0–7	34–38	63–67	0–14	38–42	63–67
	≥35	111–120	0	8–12	19–23	0	8–12	19–23
	≥35	121–130	0–7	16–20	28–32	0–7	16–20	28–32
	≥35	131–140	0–7	20–24	34–38	0–14	24–28	34–38
	≥35	141–150	0–7	24–28	43–47	0–14	27–31	43–47
	≥35	151–160	0–7	29–33	53–57	0–14	33–37	53–57

[a]This includes use of diammonium phosphate and other NP-containing sources for the early fertilizer application
[b]This includes use of single superphosphate and urea as the N and P sources for the early fertilizer application
[c]Manual transplanting with seedling ages of <25 and ≥25 days occurred only in kuruvai
[d]Manual transplanting with seedling ages of <35 and ≥35 days occurred only in samba and thaladi
[e]N application at tillering is omitted when old seedlings are used for very short duration varieties

Supplementary Table 3.2 Recommended times for three applications of fertilizer N for direct wet-seeded rice as affected by growth duration of rice variety and fertilizer source for early application of N and P

Growth duration (days)	Days after sowing					
	Compound NP fertilizer[a]			Separate sources of N and P[b]		
	Early	Tillering	Panicle initiation	Early	Tillering	Panicle initiation
81–90	0–7	16–20	28–32	0–7	16–20	28–32
91–100	0–7	20–24	34–38	12–16	24–28	34–38
101–110	0–7	25–29	44–48	12–16	28–32	44–48
111–120	0–7	29–33	54–58	12–16	33–37	53–57
121–130	0–7	34–38	63–67	12–16	38–42	63–67
131–140	0–7	39–43	73–77	12–16	42–46	73–77
141–150	0–7	44–48	83–87	12–16	47–51	83–87

[a]This includes use of diammonium phosphate and other NP-containing sources for the early fertilizer application
[b]This includes use of single superphosphate and urea as the N and P sources for the early fertilizer application

Part 4. Information included within NMR recommendations

Nutrient Manager for Rice (NMR) calculates fertilizer amounts and application dates for the rice variety and size of a field indicated by a rice farmer. NMR then enables this output to be printed and provided to the rice farmer as a personalized season- and field-specific recommendation. Examples of NMR recommendations are shown for kuruvai (Supplementary Fig. 4.1) and thaladi (Supplementary Fig. 4.2) seasons.

The name of the farmer, location, water source, crop establishment method, rice variety, sowing date, and size of the field are obtained during an interview of the farmer using NMR. The fertilizer sources for P and K (diammonium phosphate (DAP) and muriate of potash (MOP, 0-0-60) in the figures) are selected by the farmer from a list of options during the interview using NMR. In kuruvai, the recommendation indicates times for fertilizer application in days after transplanting (DAT) for both young (<25 days) and old (≥25 days) transplanted seedlings because release of irrigation water is sometimes delayed resulting in use of older rice seedlings than anticipated by the farmer during the NMR interview. In thaladi, the recommendation indicates times for fertilizer application based on the age of the seedling (<35 or ≥35 days) selected by the farmer during the NMR interview. When a farmer establishes rice by direct seeding rather than transplanting, the recommendation indicates times for fertilizer application in days after sowing.

Nutrient Manager for Rice Tamil Nadu Beta Version

Name of farmer: Arun Swami
Location: Vilangudi, Thiruvaiyaru, Thanjavur

Water source: Tube well only
Crop establishment: Manual transplanting
Variety: ADT 43
Sowing date: June 1, 2018

Note: Use this recommendation for kuruvai season 2018 only.
Target yield (total harvest): 40 bag/acre, 5.4 t/ha (14% MC)

Manage rice as shown below:

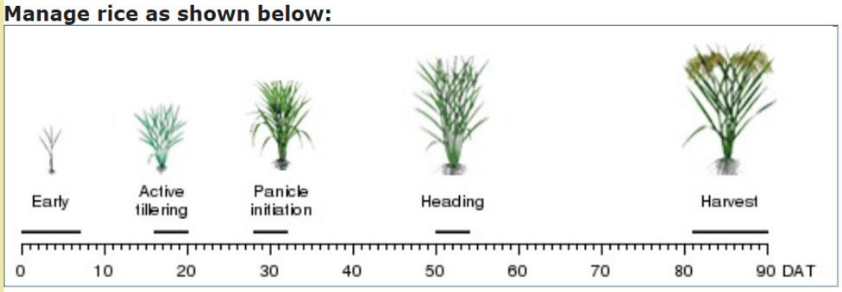

Apply fertilizer as indicated below:

Growth stage	DAT*	**A. If seedling age is younger than 25 days:** Fertilizer amount for 1 acre, 0.4 ha				DAT*	**B. If seedling age is 25 days or older:** Fertilizer amount for 1 acre, 0.4 ha			
		DAP (kg)	MOP (kg)	Urea (kg)	Zn Sulfate (kg)		DAP (kg)	MOP (kg)	Urea (kg)	Zn Sulfate (kg)
Early	0-7	28	15	26	10	0-5	28	15	26	10
Active tillering	16-20	---	---	34	---	12-16	---	---	34	---
Panicle initiation	28-32	---	15	34	---	21-25	---	15	34	---

* DAT-days after transplanting

Supplementary Fig. 4.1 An example of a Nutrient Manager for Rice (NMR) recommendation for the kuruvai season in the Cauvery Delta in Tamil Nadu, India

Nutrient Manager for Rice Tamil Nadu Beta Version

Name of farmer: Arun Swami
Location: Vilangudi, Thiruvaiyaru, Thanjavur

Crop establishment: Manual transplanting
Variety: ADT 38
Sowing date: October 1, 2018

Note: Use this recommendation for thaladi season 2018 only.
Target yield (total harvest): 41 bag/acre, 5.8 t/ha (14% MC)

Manage rice as shown below:

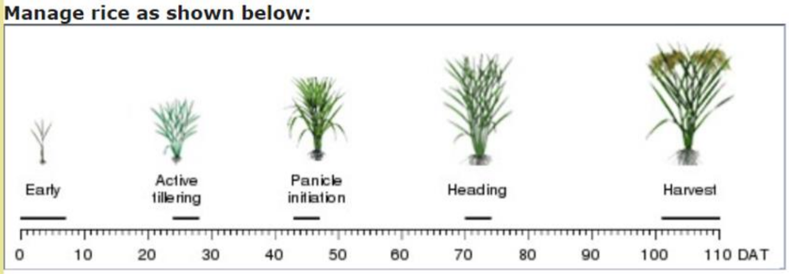

Apply fertilizer as indicated below:

Growth stage	DAT*	Fertilizer amount for 1 acre, 0.4 ha			
		DAP (kg)	MOP (kg)	Urea (kg)	Zn Sulfate (kg)
Early	0-7	27	17	16	10
Active tillering	24-28	---	---	44	---
Panicle initiation	43-47	---	17	44	---

* DAT-days after transplanting

Supplementary Fig. 4.2 An example of a Nutrient Manager for Rice (NMR) recommendation for the thaladi season in the Cauvery Delta in Tamil Nadu, India
