# Supplementary material for: Web-based tool for calculating field-specific nutrient management for rice in India
Source: Nutr Cycl Agroecosyst. 2018 Oct 22;113:21–33. doi: 10.1007/s10705-018-9959-x (PMC7357723; doi:10.1007/s10705-018-9959-x)
Supplement: Supplementary file 2 [file NCA-2019-s10705-018-9959-x-S2.pdf]

Online Resource 2. Supplementary material for field trials in Cauvery Delta, Tamil Nadu, India

Article title:
Web-based tool for calculating field-specific nutrient management for rice in India

Journal name:
Nutrient Cycling in Agroecosystems

Author names:
Sheetal Sharma, P. Panneerselvum, Rowena Castillo, Shriram Manohar, Raj Rajendran, V. Ravi, Roland J. Buresh

Author affiliations:
Sheetal Sharma, P. Panneerselvum, Shriram Manohar
International Rice Research Institute, India

Rowena Castillo, Roland J. Buresh
International Rice Research Institute, DAPO Box 7777, Metro Manila, Philippines

Raj Rajendran
Coconut Research Station, Tamil Nadu Agricultural University, Veppankulam, Thanjavour, Tamil Nadu, India

V. Ravi
Tamil Nadu Rice Research Institute, Tamil Nadu Agricultural University, Aduthurai, Thanjavour, Tamil Nadu, India

Email address of corresponding author:
Sheetal Sharma: Sheetal.sharma@irri.org

Content

Part 1. Soil properties for field trials 2
Part 2. Fertilizer N, P, and K rates for Nutrient Manager for Rice (NMR) and farmer's fertilizer practice (FFP) in field trials 3

Part 1. Soil properties for field trials

Composite soil samples were collected at 0–15 cm depth from each trial before crop establishment and fertilizer application in kuruvai and samba seasons. Soil samples were air dried and crushed. Soil pH and electrical conductivity (EC) were determined in water with a 1:2 soil–suspension ratio, organic C was determined by the Walkley–Black chromic acid wet oxidation method, available N was determined by distillation with alkaline potassium permanganate, Olsen P was determined by extraction with 0.5 M sodium bicarbonate at pH 8.5, and exchangeable (exch) K was determined by extraction with 1 M ammonium acetate at pH 7. The means and ranges in soil properties for the 14 trials in kuruvai and 40 trials in samba are shown in Supplementary Table 1.1.

Supplementary Table 1.1 Soil properties for trials in kuruvai and samba seasons in Tamil Nadu, India

Soil property	Season	Mean	Minimum	25% quartile	Median	75% quartile	Maximum
pH	Kuruvai	7.5	6.9	7.3	7.5	7.8	8.1
	Samba	6.9	5.5	6.6	7.0	7.3	7.9
EC (dS m^{-1})	Kuruvai	0.45	0.11	0.27	0.33	0.59	1.08
	Samba	0.28	0.07	0.17	0.24	0.34	0.74
OC (g kg^{-1})	Kuruvai	7.0	1.1	6.7	7.3	9.1	9.6
	Samba	5.8	0.9	3.6	5.4	8.1	10.4
N (mg kg^{-1})	Kuruvai	85	45	83	87	99	104
	Samba	77	45	62	73	94	108
Olsen P (mg kg^{-1})	Kuruvai	15	7	9	11	22	31
	Samba	15	5	9	16	20	29
Exch K (cmol kg^{-1})	Kuruvai	0.47	0.24	0.28	0.40	0.58	0.86
	Samba	0.33	0.07	0.19	0.29	0.41	0.76

Part 2. Fertilizer N, P, and K rates for Nutrient Manager for Rice (NMR) and farmer's fertilizer practice (FFP) in field trials

Rates of fertilizer N, P, and K varied more for the farmer's fertilizer practice (FFP) than Nutrient Manager for Rice (NMR) across the field trials in each season (Supplementary Table 2.1). Some farmers applied no P in samba, whereas others applied more than NMR. Similarly, some farmers applied no K in samba and thaladi, whereas others applied more than NMR. The fertilizer rates for the blanket fertilizer recommendation (BFR) (not shown in Supplementary Table 2.1) were a constant 150 kg N ha^{-1}, 22 kg P ha^{-1}, and 42 kg K ha^{-1} for all trials in all seasons.

Supplementary Table 2.1 Ranges in rates of fertilizer N, P, and K applied through Nutrient Manager for Rice (NMR) and farmer's fertilizer practice (FFP) in three rice-growing seasons in Tamil Nadu, India

Nutrient	Season	Treatment	Nutrient rate (kg ha^{-1})				
			Minimum	25% quartile	Median	75% quartile	Maximum
N	Kuruvai	NMR	103	115	121	123	143
		FFP	73	102	113	138	147
	Samba	NMR	121	122	124	141	156
		FFP	40	96	112	124	275
	Thaladi	NMR	115	125	131	132	142
		FFP	79	112	123	143	230
	All	NMR	103	122	124	136	156
		FFP	40	101	114	137	275
P	Kuruvai	NMR	12	14	14	14	16
		FFP	9	18	25	25	27
	Samba	NMR	14	14	15	17	18
		FFP	0	12	22	25	50
	Thaladi	NMR	10	12	13	13	15
		FFP	9	20	25	25	36
	All	NMR	10	14	14	15	18
		FFP	0	13	25	25	50
K	Kuruvai	NMR	20	25	30	39	40
		FFP	25	31	42	59	65
	Samba	NMR	24	48	50	58	68
		FFP	0	30	56	63	150
	Thaladi	NMR	23	24	27	37	53
		FFP	0	30	31	56	86
	All	NMR	20	29	48	50	68
		FFP	0	31	49	63	150

Number of field trials was 14 in kuruvai, 40 in samba, and 20 in thaladi
